# Recruitment into the Alzheimer Prevention Trials (APT) Webstudy for a Trial-Ready Cohort for Preclinical and Prodromal Alzheimer’s Disease (TRC-PAD)

**DOI:** 10.14283/jpad.2020.46

**Published:** 2020

**Authors:** S. Walter, T.B. Clanton, O.G. Langford, M.S. Rafii, E.J. Shaffer, J.D. Grill, G.A. Jimenez-Maggiora, R.A. Sperling, J.L. Cummings, P.S. Aisen, TRC-PAD Investigators

**Affiliations:** 1. Alzheimer’s Therapeutic Research Institute, University of Southern California, San Diego, CA, USA;; 2. Center for Alzheimer Research and Treatment, Brigham and Women’s Hospital, Harvard Medical School, Boston, MA, USA;; 3. Institute for Memory Impairments and Neurological Disorders, University of California, Irvine;; 4. Department of Brain Health, School of Integrated Health Sciences, University of Las Vegas, Nevada; Cleveland Clinic Lou Ruvo Center for Brain Health, USA

**Keywords:** Trial-ready cohort, online registry, remote recruitment, web-based, preclinical, Alzheimer’s disease, prevention.

## Abstract

**BACKGROUND:**

The Alzheimer Prevention Trials (APT) Webstudy is the first stage in establishing a Trial-ready Cohort for Preclinical and Prodromal Alzheimer’s disease (TRC-PAD). This paper describes recruitment approaches for the APT Webstudy.

**OBJECTIVES:**

To remotely enroll a cohort of individuals into a web-based longitudinal observational study. Participants are followed quarterly with brief cognitive and functional assessments, and referred to Sites for in-clinic testing and biomarker confirmation prior to enrolling in the Trial-ready Cohort (TRC).

**DESIGN:**

Participants are referred to the APT Webstudy from existing registries of individuals interested in brain health and Alzheimer’s disease research, as well as through central and site recruitment efforts. The study team utilizes Urchin Tracking Modules (UTM) codes to better understand the impact of electronic recruitment methods.

**SETTING:**

A remotely enrolled online study.

**PARTICIPANTS:**

Volunteers who are at least 50 years old and interested in Alzheimer’s research.

**MEASUREMENTS:**

Demographics and recruitment source of participant where measured by UTM.

**RESULTS:**

30,650 participants consented to the APT Webstudy as of April 2020, with 69.7% resulting from referrals from online registries. Emails sent by the registry to participants were the most effective means of recruitment. Participants are distributed across the US, and the demographics of the APT Webstudy reflect the referral registries, with 73.1% female, 85.0% highly educated, and 92.5% Caucasian.

**CONCLUSIONS:**

We have demonstrated the feasibility of enrolling a remote web-based study utilizing existing registries as a primary referral source. The next priority of the study team is to engage in recruitment initiatives that will improve the diversity of the cohort, towards the goal of clinical trials that better represent the US population.

## Background

Identifying eligible participants for early intervention Alzheimer’s disease (AD) clinical trials continues to be a significant challenge in the field (1, 2). The overarching aim of the Trial-Ready Cohort in Preclinical and Prodromal Alzheimer’s Disease (TRC-PAD) program is to accelerate enrollment for early stage AD clinical trials (3). This will be accomplished by identifying and screening participants to confirm eligibility for these trials, including amyloid biomarker confirmation, and then monitoring and maintaining engagement with these participants through longitudinal visits until an appropriate trial is available. The considerations behind the design of TRC-PAD are described by Aisen et al. (4). The first step in establishing the Trial-ready Cohort (TRC) was to recruit participants into the Alzheimer Prevention Trials (APT) Webstudy, an online assessment tool designed to serve as a feeder to the in-person TRC-PAD cohort. We projected the APT Webstudy would require between 25,000 and 50,000 participants, with at least 20% participants from under-represented communities, in order to identify enough eligible participants for a planned TRC of n=2,000. The APT Webstudy program requires secure and scalable informatics infrastructure (5), as well as an algorithm to identify participants and rank them by risk of brain amyloidosis and development of AD dementia (6). These elements of the program are described in separate papers in this series.

The APT Webstudy was launched as clinical trials have increasingly utilized web-based tools, including registries, to improve efficiency in screening (7–9). Although leveraging registries to recruit for clinical trials is not a new concept, the establishment of online registries has broadened access to participants who are interested and eligible for studies (10–13). Going further than remote recruitment, Orri et al (14) conducted the first entirely web-based clinical trial run under an Investigational New Drug (IND) application. Digital tools allow researchers to optimize the use of mobile technologies in clinical trials, respond to the preferences of participants (15), and measure and finetune communication methods (16). To our knowledge, TRC-PAD is the first program inviting participants from various existing registries to a join a longitudinal Webstudy with identification and referral of high-risk individuals to an in-person TRC. In this article, we describe the preliminary experience of efforts to recruit to APT Webstudy, including from national and local registries, as a unifying path to enrollment in TRC-PAD.

## Methods

### APT Webstudy Experience

Participants log in using either their existing social login credentials or by creating an account and providing a username, email address and password. Once logged on, participants are considered ‘registered.’ The Webstudy is designed as a ‘walk through’ experience, with each new section opening after completion of the former. The sections are: Step 1: Personal Profile; Step 2: Consent; Step 3 Lifestyle; Step 4: Remote cognitive and functional assessments; Step 5: Review scores. Sections are described in more detail in a separate paper in this series (17). The questionnaires and assessments were designed to be brief with a target duration of 15 minutes.

### Recruitment

APT Webstudy participants are recruited from multiple sources. For the purposes of this paper, the term registry refers to a online registry, study, or service matching individuals interested in participating in studies or clinical trials to prevent or delay AD dementia. Early in its development, the TRC-PAD study team established partnerships with each of the largest “Feeder” registries, and in collaboration with the managing team or investigators, developed a referral strategy based on the registry’s unique population and existing communication pathways. Each strategy began small and was expanded when we were able to ensure the stability of the Webstudy infrastructure, as well as our capacity to provide user support. Outreach took the form of direct email campaigns highlighting the APT Webstudy on the registry website, e-newsletters, and social media posts. In addition to referrals from registries, both central and site-based strategies were employed.

### UTM Codes

Urchin Tracking Modules (UTM) were generated to track participants that registered for the APT Webstudy in response to digital outreach, and were embedded in emails, webpages, and social media advertisements. For some registries, although various outreach activities were utilized, all responses linked back through the registry website, requiring use of a single UTM, and limiting our ability to understand the response rates to different digital communications. Recruitment strategies that did not utilize a UTM included printed materials (i.e., brochures, newsletters and magazines) and earned media (i.e., online and print newspaper articles).

### The Alzheimer’s Prevention Registry (APR) (www.endalznow.org)

**Figure 1.**
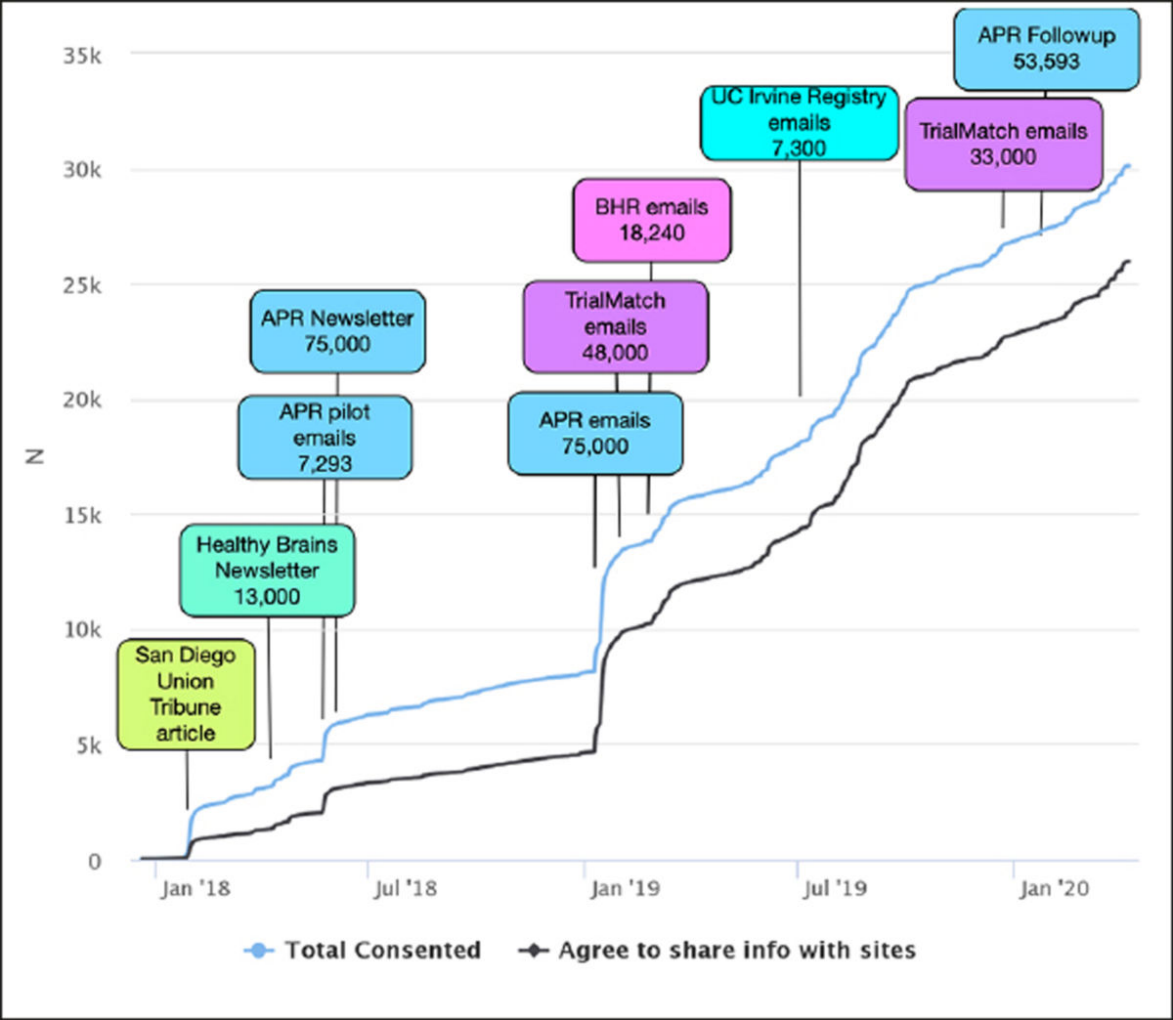
Alzheimer Prevention Trials (APT) Webstudy: Feeder Registry Recruitment Campaign Timeline

APR was launched in October 2012 by the Banner Alzheimer’s Institute with the aim of providing a shared resource to the AD scientific community to facilitate enrollment in studies to prevent AD. In 2015, APR began offering an optional APOE genotyping program (GeneMatch) to members ages 55–75 to help match individuals to research studies. As of August 2018, APR enrolled a total 320,000 participants with 75,351 agreeing to the GeneMatch program, and approximately 75,000 agreeing to be contacted by researchers (18). APR participants are primarily women (65.6%) and Caucasian (45.5%); 1.8% are Hispanic/Latino and less than 1% are from other underrepresented groups. It should be noted that these percentages are a reflection of only the 60.8% of APR participants who provided their Race or Ethnicity (Table 1) (19). 14% of APR participants are age 50–59, 35% age 60–69, and 23% age 70–79 (Table 1). The APT Webstudy recruitment strategy began with a pilot phase in April 2018, with batches of emails sent from APR to 7,293 individuals (Figure 1). This was followed by an article in the APR quarterly newsletter introducing the APT Webstudy and posts on APR’s social media accounts. In January 2019, emails were sent in batches to 75,000 registrants inviting them to join the APT Webstudy. In March and April 2020, follow up emails were sent to participants who had not opened the email or clicked the link for the APT Webstudy, with additional reminders scheduled for May 2020.

### Alzheimer’s Association TrialMatch (trialmatch.alz.org)

Alzheimer’s Association TrialMatch (trialmatch.alz.org) is a free online matching service that utilizes user’s information to generate a custom report of clinical trials for which they may be a good fit. TrialMatch has a large pool of 322,997 users, with 134,148 providing contact and personal information. Individuals enrolled in TrialMatch indicate whether they are a healthy volunteer (52.8%), a caregiver looking for clinical trials for someone else such as a family member with AD (31.7%), or a person living with the disease looking for trials (13.3%). A small percent (2.2%) of users are entered into TrialMatch by a physician or researcher. Individuals under 50 comprise 35% of the Healthy Volunteers and 20% of all TrialMatch participants. 69% of TrialMatch are over the age of 50. Participants are 73.4% Caucasian, 4.5% Hispanic/Latino, and 65% are women. Women comprise 78% of the healthy controls and 54% of caregivers looking for trials for someone else. 22% of TrialMatch users either care for someone with a diagnosis of AD or have a diagnosis of AD. The first APT Webstudy recruitment campaign began in March 2019, with direct emails targeting 48,000 TrialMatch users living within 200 miles of potential TRC-PAD clinical sites. An additional 33,000 users were invited to join APT Webstudy beginning in December 2019. Emails were sent in batches of 5,000 twice a week, and is ongoing at the time of this manuscript.

### The Brain Health Registry (BHR) (brainhealthregistry.org)

The Brain Health Registry (BHR) (brainhealthregistry.org) collects longitudinal health, cognitive, and lifestyle data through detailed self-report questionnaires and online cognitive tests (Cogstate, Lumosity, and MemTrax) (16). BHR was launched in 2014 and currently has baseline data on 56,982 participants. BHR participants are 80.9% Caucasian, 5.3% Hispanic/Latino, 73.9% women, with 73% of participants over the age of 50 (20) (Table 1). The BHR team sent emails to 18,240 participants inviting them to register for the APT Webstudy beginning in March 2019 (Figure 1). Emails were sent in batches of 500 every week. If participants do not respond, two follow-up emails are sent, with a second set of reminder emails 231 and 238 days from their initial email contact. The BHR team also featured the APT Webstudy in their e-newsletter.

### The Cleveland Clinic Healthy Brains Registry (healthybrains.org)

The Cleveland Clinic Healthy Brains Registry (healthybrains.org) is a longitudinal, web-based symptomatic and lifestyle assessment (21), with over 13,000 registrants, and over half expressing interest in enrolling into clinical trials. HealthyBrains has registrants and newsletter subscribers from across the nation. The highest number of registrants in the US states of Ohio, Nevada, California and Florida. Registrants were invited to join the APT Webstudy through an article on the HealthyBrains website in May 2018, followed by features in two newsletters, sent by email (Figure 1).

### UCI Consent-to-Contact (C2C) Registry (c2c.uci.edu)

UCI Consent-to-Contact (C2C) Registry (c2c.uci.edu) is a confidential online tool to help match local volunteers in Orange County, CA, with research studies at the University of California, Irvine (22). Registrants enroll by providing an email address or by phoning the research site, remotely completing a series of questions regarding medical history and research interests. Beginning in July 2019, 7,300 C2C participants were invited by email to join the APT Webstudy (Figure 1).

### Other sources

Anticipating that the registry-based approach would have limitations, especially in identifying eligible participants from under-represented groups, the APT Webstudy team developed recruitment strategies utilizing the TRC-PAD site network as well as other central activities. Sites participating in the TRC-PAD cohort study were identified early in the development of the program, with some agreeing to work locally to recruit participants to the APT Webstudy. Each of the TRC sites were invited to utilize their own databases of individuals interested in clinical research and email information about the APT Webstudy. The TRC-PAD study team provided flyers, postcards, newsletter and email template language, social media content and leaflets describing the APT Webstudy. Language for these materials was approved by the Institutional Review Board (IRB) and UTM codes were generated where appropriate. Sites also held community outreach events, partnered with other local community organizations to share information about the study, advertised on social media, and posted information about the Webstudy on their own webpages. Central recruitment efforts included generating earned media including newspaper and online and print edition magazine articles, local TV interviews, and posting the study on websites for clinical trials and AD. The earned media stories included an article in the San Diego Union Tribune in January 2018, two letters to the editor in May 2019, in local papers that have circulations of 80,000 (Charleston, SC) and 150,000 (Lexington, KY) respectively. Grand Magazine published an online piece about the APT Webstudy on August 12, 2019, generating 54,000 impressions. The Saturday Evening Post, with a circulation of 302,000 and majority of readers over the age of 45, included APT in its January/February 2020 print edition. So far, the only paid advertising was in the form of Facebook advertisements. Facebook ads ran in eight markets for two weeks in November 2018 for a cost of $12,000, and six markets for 5 weeks in August-September 2019 for a cost of $3,000. The ads were targeted geographically and to the largest minority population in each location, based around the location of TRC sites.

## Results

APT Webstudy Enrollment: At the time of preparing this mansuscript, there are 30,650 participants consented to the APT Webstudy. Recruitment strategies for the first year were a mix of central and local efforts (Figure 1). The first notable increase was in January 2018 following local newspaper coverage. In March 2018, email referrals were piloted for APR Registry. In April 2018, APR and HealthyBrains introduced the Webstudy in their newsletters. In the first year, 388 participants per month consented to the APT Webstudy on an average. The APR email referrals began in earnest in January 2019, leading to a dramatic increase in consented participants, with 5,196 consenting in January 2019 (Figure 1). This was followed by email referrals from TrialMatch and BHR. In the second year, participants consented to the APT Webstudy on an average of 1,514 per month.

### Demographics

Participants in the APT Webstudy have a mean age of 64.56 with a majority of participants ages 50–59 (28.9%) and 60–69 (44.1%) (Table 1). Most participants identify as women (73.0%), white (92.5%) and more than high school level education (85.0%). 2.3% of APT Webstudy participants self describe as Hispanic/Latino. Although most participants are retired or not working (53.2%), a significant percentage are employed either full (30.6%) or part-time (14.7%) (Table 2). A majority of participants have a family history of AD (62.6%) and do not have a personal diagnosis of AD (94.6%). Further details on lifestyle and medical history are provided on Tables 2 and 3.

### Enrollment by Referral sources

At this point in the recruitment to the APT Webstudy, registries were the primary source of participants, with referrals resulting in 69.69% of consented individuals, according to UTM codes. APR was by far the biggest contributer with 38.98% of all APT Webstudy consented participants, followed by 25.40% referred by TrialMatch. Those referred by APR were also slightly more likely to both register and consent to APT (Table 3). All together 15.9% of the APR participants that were contacted consented to APT, compared to 9.8% or less for other registries. Email (32.92%) and websites (40.78%) were the most common mode of referral, however website visits were largely driven by email campaigns. Central media efforts that could be tracked with UTM resulted in 234 participants. The central Facebook ads accounted for 7,800 and 3,000 clicks which translated to 0.15% of consenting participants.

### Geographic Distribution

**Figure 2.**
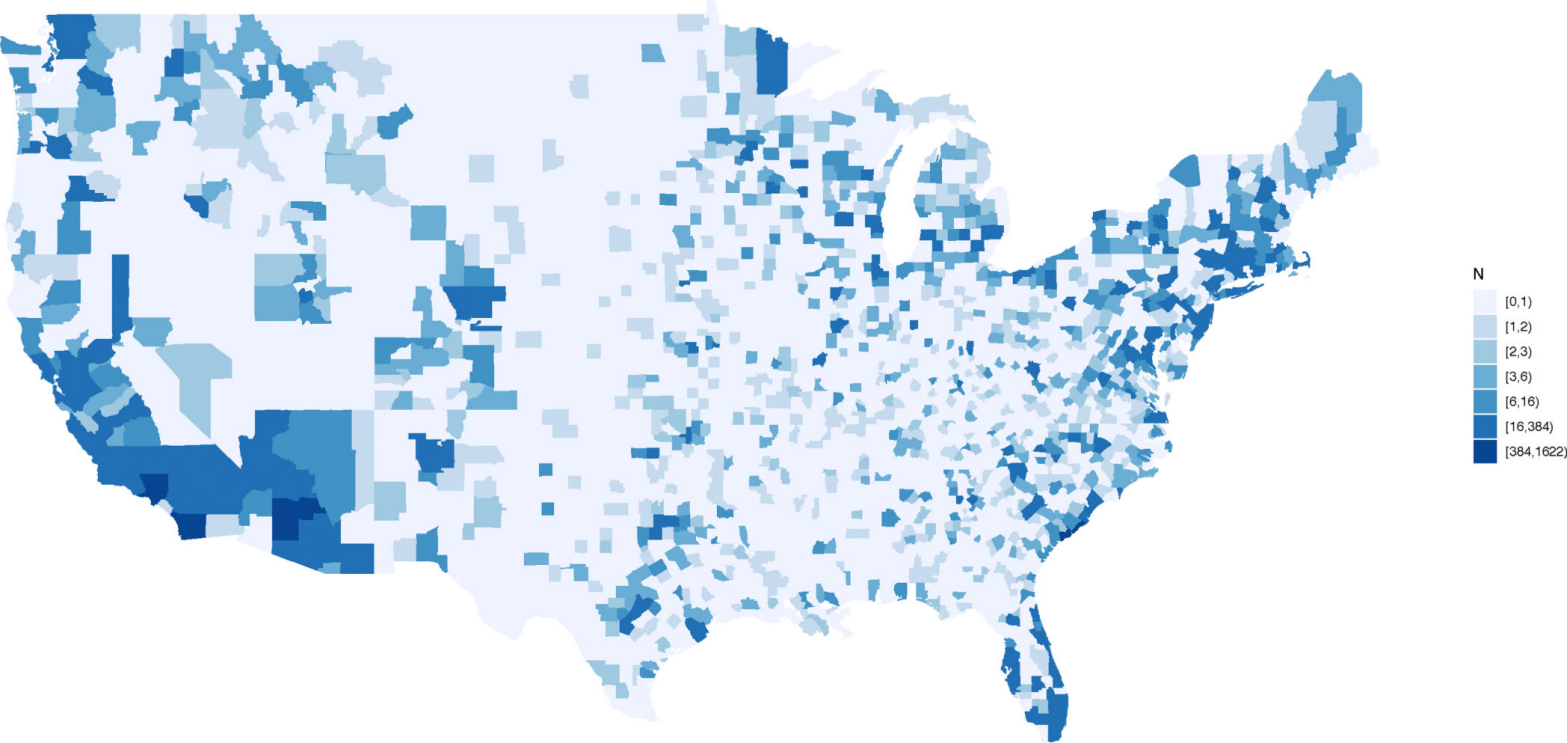
APT Webstudy Enrollment: Heatmap of US Counties

APT Webstudy participants reside in each of the 50 United States (US), the District of Columbia, and Puerto Rico. States with the highest number of consented participants include California (16.63%), Florida (5.65%), New York (4.67%), Texas (4.66%), and Virginia (4.38%). International location is not currently collected. Participants consented to the APT Webstudy reside in 1931 (or 60%) of US counties. The top ten counties with participants consented to APT are San Diego County, CA (n=1621); Orange County, CA (n=861) Maricopa County, AZ (n=764), Los Angeles County, CA (n=612), Cook County, IL (n=443) Charleston County, SC (n=384), Fayette County, KY (n=279), King County, WA (n=270) Pima County, AZ (n=239) and Middlesex County, MA (n=238) (Figure 2).

## Discussion

We have demonstrated that online registries are not only feasible but they are an excellent method to identify and recruit participants for a Webstudy. Participants in a registry have already demonstrated an interest in research and willingness to provide information about themselves. In addition, registries have communication infrastructure and digital platforms designed to engage individuals through educational materials, newsletters and other outreach, which may lead to higher rates of referral. UTM codes were shown to be an effective method to track the referral source in this study. The strategy that yielded highest rates of responses was to first feature the APT Webstudy in the registry’s newsletter, followed by direct email communication to registrants. Although not tracked with separate UTM codes, the consistent increase of participants demonstrates that sending second and third emails to non-responders produces additional participants. Although central media efforts and social media advertising were piloted in this first stage of recruitment, this strategy has not been fully explored as a potential source for remotely enrolled participants.

The registries used in this study had a contact-to-consent rate ranging from 1.8%–15.9%, despite having very similar composition of registrants. This brings up several questions as to best practices. Was the higher rate of consent from APR compared to BHR due to the fact that APR directly targets individuals interested in clinical trials? Could the observed rate of consent to contacted participant be influenced by the level of engagement utilized by the respective registries?

It is not surprising that the demographics of participants in the APT Webstudy are similar in demographics to the registries that referred the majority of participants. However, understanding why such a large majority of participants are women is important. Further research may reveal both barriers to in-person research and preferences for online studies. The low rate of Hispanic/Latino involvement in APT Webstudy can likely be attributed to 2 factors, (1) the low rates of Hispanic/Latino participants in the referral registries and (2) the APT Webstudy and recruitment materials had not been translated into Spanish.

We acknowledge that the APT Webstudy has an inherent selection bias, in that participants must have access to the internet in order to participate. This disproportionately excludes many people from under-represented communities, where according to recent Pew reports, only 57% of Hispanic and African American adults own a laptop or a tablet (23), compared to 82% of Caucasians. Although those over 65 years of age are more likely to use a desktop or tablet to access the internet, lower income Americans, those with less than college education, and black and Hispanic populations, are all more likely to use a cell phone to access the internet (24). Although the APT Webstudy is mobile-friendly, the cognitive testing at present requires use of a tablet or computer. The study team is considering changes to cognitive testing that will allow for the use of smart phones and expand accessibility to all communities. Other researchers (25) have demonstrated that text messages can be an effective communication channel with research participants. Would people be more responsive to a text message inviting them to return for a study visit?

The Spanish language version of the APT Webstudy was launched early in 2020, with efforts underway to optimize the cultural sensitivity of the Webstudy and all participant-facing content. A key aim of the study is to engage in recruitment initiatives that will improve the diversity of the cohort, towards the goal of clinical trials that better represent the US population. For the African-American community in particular, recruitment campaigns will highlight disparities in Alzheimer’s disease risk and care, and the role research and clinical trials can play in effecting change.

This study has several limitations. The feeder registries differ in numerous ways, including sample sizes, aims or purpose, geographic distribution, length of time from when participants were first engaged with, and frequency of participant engagement. The current analyses did not account for these differences. Similarly, varying levels of data were available for participants in feeder registries, preventing combination of data streams for more sophisticated analyses of recruitment efficiency. Recruitment from feeder registries was peformed over multiple years, introducing potential confounding by time. Quantification of site level efforts toward recruitment was minimal, limiting our ability to understand the efficacy of site level efforts relative to using central efforts or these feeder registries.

In conclusion, this study demonstrates the feasibility of recruiting from feeder registries into a common platform for identifying potentially eligible participants for a Trial-ready cohort. A robust sample was assembled in a relatively short period of time that is anticipated to play a key role in the national AD clinical trial agenda.

## Figures and Tables

**Table 1.** Feeder Registries and APT Demographics

	Alzheimer’s Prevention Registry (APR) Nov 2019 (15)	Brain Health Registry (BHR) Jan 2018 (16)	TrialMatch Jan 2020	APT Webstudy Apr 20, 2020
	*Consented*			
	346,655	56,982	134,148	30,650
	*Age*			
Below 50	31,648 (9.12%)	15,772 (27.7%)	27,861 (20.77%)	357(1.16%)
50–59	47,443 (13.69%)	14,504 (25.5%)	26,273 (19.59%)	8,886 (28.99%)
60–69	120,794 (34.85%)	17,255 (30.3%)	29,625 (22.08%)	13,533 (44.15%)
70–79	79,934 (23.05%)	8,285 (14.5%)	24,273 (18.09%)	6,814 (22.23%)
80–89	13,312 (3.84%)	1,611 (2.8%)	12,906 (9.62%)	967 (3.15%)
Over 90	2,253 (0.65%)		1,489 (1.11%)	39 (0.13%)
Missing	51,271 (14.8%)	not reported	11,721 (8.74%)	54 (0.18%)
Mean Age	63.3	55.0	64.76	64.56
	*Gender*			
Male	75,853 (21.9%)	14,865 (26.1)	33,394 (24.89%)	8,212 (26.8%)
Female	227,559 (65.6%)	42,117 (73.9%)	87,081 (64.91%)	22,389 (73.0%)
Missing	43,243 (12.5%)	not reported	18,019 (13.43%)	(0.2%)
	*Race and Ethnicity*			
Asian	2,074 (0.60%)	1,810 (3.2%)	2,348 (1.75%)	432 (1.4%)
African-American	4,149 (1.20%)	2,584 (4.5%)	4,245 (3.16%)	474 (1.5%)
American Indian or Alaska Native	(>0.5%)	242 (0.4%)	639 (0.48%)	67 (0.2%)
Hawaiian/Pacific Islander	(>0.5%)	104 (0.2%)	185 (0.14%)	39 (0.1%)
Hispanic or Latino	6,223 (1.80%)	3,041 (5.3%)	6,046 (4.51%)	711 (2.3%)
White	157,660 (45.48%)	46,109 (80.9%)	98,449 (73.39%)	28,363 (92.5%)
Other, prefer not to answer	35,266 (10.17%)	2,715 (4.7%)	2,249 (1.67%)	457 (1.5%)
Missing	139,208 (40.16%)	1,367 (2.4%)	24,444 (18.22)	107 (0.3%)
	*Education level*			
Advanced Degree	not reported	18,893 (33.2%)	29,637 (22.09%)	12,018 (39.2%)
College or some college	not reported	31,676 (55.59%)	63,574 (47.39%)	14,030 (45.8%)
High School or less	not reported	6,413 (11.3%)	22,918 (17.08%)	4,371 (14.3%)
Prefer not to answer	not reported	not reported	not reported	136 (0.4%)
missing	not reported	not reported	18,019 (13.43%)	95 (0.3%)
	*Diagnosis of Alzheimer’s disease?*			
Yes	9,550 (2.7%)	251 (0.4%)	29,663 (22.11%)	1,398 (4.6%)
No	149,619 (43.2%)	not reported	NA	28,999 (94.6%)
Missing	187,486 (54.1%)	not reported	NA	253 (0.8%)
	*Parent or sibling diagnosed with Alzheimer’s disease or dementia?*			
Yes	70,098 (20.2%)	14,267 (25.0%)	not reported	19,201 (62.6%)
No	28,039 (8.1%)	not reported	not reported	10,976 (35.8%)
Prefer not to answer	42,058 (12.1%)	not reported	not reported	247 (0.8%)
Missing	206,460 (59.6%)	not reported	not reported	226 (0.7%)

**Table 2.** APT Webstudy Health and Lifestyle

*Employment Status*	
Retired/not working	16,319 (53.2%)
Full time	9,375 (30.6%)
Part time	4,513 (14.7%)
Prefer not to answer	213 (0.7%)
Missing	230 (0.8%)
*Exercise regularly (one hour per week)*	
Yes	22,784 (74.3%)
No	7,514 (24.5%)
Prefer not to answer	128 (0.4%)
Missing	224 (0.7%)
*Drinks alcohol regularly (2 drinks per day or more)*	
Yes	5,419 (17.7%)
No	24,789 (80.9%)
Prefer not to answer	156 (0.5%)
Missing	286 (0.9%)
*Preferred contact method*	
Email	24,145 (78.8%)
Phone call	941 (3.1%)
No preference	1,401 (4.6%)
Missing	4,163 (13.6%)
*Medical History*	
Diabetes	2,682 (8.75%)
High Blood Pressure	10,228 (33.47%)
Vascular Disease	1,320 (4.31%)
None of the above	15,192 (49.57%)
Prefer not to answer	53 (0.17%)

**Table 3.** APT Webstudy Recruitment by Referral Source

Referral Source	Contacted (approx)	Consented to APT	Registered and no consent APT	% of APT consented	Rate of consent to registered	% Contacted that consented
Registry						
APR	75,000	11,947	555	38.98%	95.56%	15.93%
TrialMatch	81,000	7,786	854	25.40%	90.12%	9.61%
UCI C2C Registry	7,300	716	69	2.34%	91.21%	9.81%
BHR	18,240	668	67	2.18%	90.88%	3.66%
HealthyBrains	13,000	242	33	0.79%	88.00%	1.86%
Registry Total	194,540	21,359	1,578	69.69%	93.12%	10.98%
Central with UTM		234	41	0.76%	85.09%	
Site with UTM		551	69	1.80%	88.87%	
Total participants consented with UTM		22,144		72.25%		
Total participants consented without UTM		8,506		27.75%		
Total participants consented to APT		30,650		100.00%		
